# Virtual reality among the elderly: a usefulness and acceptance study from Taiwan

**DOI:** 10.1186/s12877-019-1218-8

**Published:** 2019-08-19

**Authors:** Shabbir Syed-Abdul, Shwetambara Malwade, Aldilas Achmad Nursetyo, Mishika Sood, Madhu Bhatia, Diana Barsasella, Megan F. Liu, Chia-Chi Chang, Kathiravan Srinivasan, Raja M., Yu-Chuan Jack Li

**Affiliations:** 1 International Center for Health Information Technology (ICHIT), Taipei Medical University, Taipei, Taiwan; 2 Graduate Institute of Biomedical Informatics, Taipei Medical University, Taipei, Taiwan; 3 Chitkara University, Punjab, India; 4 I.K. Gujral Punjab Technical University, Kapurthala, Punjab India; 5 School of Gerontology Health Management, College of Nursing, Taipei Medical University, 250 Wuxing Street, Taipei, Taiwan 11031 People’s Republic of China; 6 College of Interdisciplinary Studies, Taipei Medical University, Taipei, Taiwan; 7 School of Information Technology and Engineering, Vellore Institute of Technology (VIT), Vellore, India; 8 TMU Research Center of Cancer Translational Medicine, Taipei Medical University, Taipei, Taiwan; 9 Tasikmalaya Polytechnic of Ministry of Health, Tasikmalaya, West Java Indonesia

**Keywords:** Virtual reality, Active aging, Older people, Technology acceptance model

## Abstract

**Background:**

Virtual reality (VR) has several applications in the medical domain and also generates a secure environment to carry out activities. Evaluation of the effectiveness of VR among older populations revealed positive effects of VR as a tool to reduce risks of falls and also improve the social and emotional well-being of older adults. The decline in physical and mental health, the loss of functional capabilities, and a weakening of social ties represent obstacles towards active aging among older adults and indicate a need for support. Existing research focused on the effects of VR among older populations, and its uses and benefits. Our study investigated the acceptance and use of VR by the elderly.

**Methods:**

This pilot study was conducted on 30 older adults who voluntarily participated during March to May 2018. Nine VR applications that promote physical activities, motivate users, and provide entertainment were chosen for this study. Participants were asked to use any one of the applications of their choice for 15 min twice a week for 6 weeks. At the end of 6 weeks, participants were asked to fill out a questionnaire based on the Technology Acceptance Model and a literature review, to evaluate their acceptance of VR technology. Cronbach’s alpha reliability analysis was used to test the internal consistency of the questionnaire items. Pearson’s product moment correlation was used to examine the validity of the questionnaire. A linear regression and mediation analysis were utilized to identify relationships among the variables of the questionnaire.

**Results:**

In total, six male and 24 female participants aged 60~95 years volunteered to participate in the study. Perceived usefulness, perceived ease of use, social norms, and perceived enjoyment were seen to have had significant effects on the intention to use VR. Participants agreed to a large extent regarding the perceived usefulness, perceived enjoyment, and their experience of using VR. Thus, VR was seen to have high acceptance among this elderly population.

**Conclusions:**

Older people have positive perceptions towards accepting and using VR to support active aging. They perceived VR to be useful, easy to use, and an enjoyable experience, implying positive attitudes toward adopting this new technology.

**Electronic supplementary material:**

The online version of this article (10.1186/s12877-019-1218-8) contains supplementary material, which is available to authorized users.

## Background

### Background of the study

The World Health Organization (WHO) projects that the global population will become a super-aged society by 2030 [1]. Currently, 12.5% of people in the world are aged 60 years or older, which is referred to an old society [1], while 24% of Taiwan’s population will be in the older age group by 2030 [2]. Declining mortality rates have been national success stories for the well-being of societies, but they come with consequences of rising morbidity [3, 4]. A study revealed that declines in physical and mental health, the loss of functional capabilities, and a weakening of social ties represent obstacles to active aging among institutionalized older adults [5]. In addition, life satisfaction among older people, with reduced self-care capacity and physical inactivity (PI), was found to be related to several factors, with social, physical, and mental aspects, such as interacting with each other; especially feeling lonely, the degree of self-care capacity, poor overall health with sleep-wake disturbances, a worried feeling, and poor financial resources [6]. The elderly also tend to have a poor quality of life [7] and develop mobility limitations [8] and functional disabilities [4].

Physical activity is seen to play an important role in improving the functional status, psychological status, and well-being, and has social benefits [9]. Several interventions to improve the physical and cognitive functioning among older people were reported [10–12]. Also, gaming interventions such as virtual environments [13, 14], Pokémon Go [15, 16], and Nintendo Wii™ [17] are being used as supporting programs for older populations. One such emerging technology is virtual reality (VR), a three-dimensional computer-generated world wherein a person can move about and interact, as if one is actually present in the imaginary space [18]. VR currently has several applications in the medical domain [18], and it also generates a secure environment to carry out certain activities [19, 20]. Evaluations of the effectiveness of VR among older populations revealed positive results for VR as a tool to reduce risks of falls [21–24], and high adherence was also observed for home-based VR settings [25] and cognitive rehabilitation [26]. Moreover, it is seen as being well-tolerated by older populations and can stimulate autobiographical memories during reminiscence therapy [27]. VR interventions have shown positive effects on the social and emotional well-being of older adults [28]. Existing research focused on the effects of VR among older populations, and its uses and benefits. However, there is limited research on the acceptance of VR among older adults and how they perceive it.

Despite the benefits offered by VR-based rehabilitation technologies, attitudes among elderly people toward using VR are still unclear [29]. Our study investigated the acceptance and use of VR among an older age group. The Technology Acceptance Model (TAM) was used to assess different factors affecting the acceptance of VR.

### Research model

The TAM was introduced by Davis (1986) and was based on the Theory of Reasoned Action (TRA). The TRA postulates that one’s behavior is directly influenced by behavioral intentions which are based on one’s attitudes and subjective norms toward a given behavior [30, 31]. The TAM illustrates a user’s acceptance of a new technology [30, 32]. The model has been extensively used in different research fields to anticipate user behaviors. It provides a theoretical basis to help explain relationships among various external variables, perceived usefulness (PU), perceived ease of use (PEOU), attitudes, intention to use (IU), and actual use [30, 33, 34]. Therefore we used the TAM to assess these factors based on a TAM scale developed by Davis [30]. We also included ‘social norms’ (SNs) [35] and ‘perceived enjoyment’ (PE) from Venkatesh [36, 37].

Davis proposed that PU is ‘the degree to which one presumes that using a specific system would augment his or her job performance’. In our study, PU was defined as ‘the degree to which older people presume that use of VR would augment their daily activities’. Also, according to Davis, PEOU refers to ‘the degree to which one accepts that using a specific system would be free of effort’ [38], and in our study, PEOU was defined as the degree to which older people perceived that using VR was free of effort. According to Davis and other studies, PU and PEOU are important factors in the acceptance of a technology [38, 39]. Thus, in order to verify if the relation between the influence of PEOU and PU in regards to VR acceptance can be applied to this study, we proposed the following hypotheses:

- Hypothesis 1 (H1): Perceived usefulness will influence the intention to use VR;
- Hypothesis 2 (H2): Perceived ease of use will influence the intention to use VR; and
- Hypothesis 3 (H3): Perceived ease of use will influence the perceived usefulness of VR.

SNs are defined as ‘a person’s perception that most people who are vital to him, think that he should or should not perform the behavior in question’ [40] and in our study, SNs were defined as ‘older adults’ perception that people who are important to them think that they should or should not use VR’. As per previous research, SNs are seen to influence the intention to use (IU) a technology [41, 42]. Thus, we formulated a hypothesis to verify this in the case of VR:

- Hypothesis 4 (H4): Social norms influence the intention to use VR.

According to Van der Heiden, in hedonic systems, “enjoyment” is an important determinant in the intention to use something [43], and Heerink et al. indicated that enjoyment is also a factor that influences the intention to use something [44]. “Perceived enjoyment” (PE) has been defined as ‘the degree to which using a technology is perceived as fun’ [36, 45]. As VR is also partly hedonic, enjoyment could be a factor that should be included in an analysis of acceptance among older adults. Thus, in our study, PE was defined as the degree to which VR was perceived as fun, and the related hypothesis was:

- Hypothesis 5 (H5): Perceived enjoyment influences the intention to use VR.

User experience is a concept covering the physical and psychological reactions or behaviors of the user before, during, and after using a technology or a product [46]. Ning and Kim demonstrated that user’s experience has an effect on usage intentions through perceived utility [47]. In our study, user experience (UE) was the behavior of older participants after using VR. We formulated two hypotheses as:

- Hypothesis 6 (H6): User experience has an effect on perceived usefulness; and
- Hypothesis 7 (H7): User experience has an effect on the perceived ease of use.

**Fig. 1:**
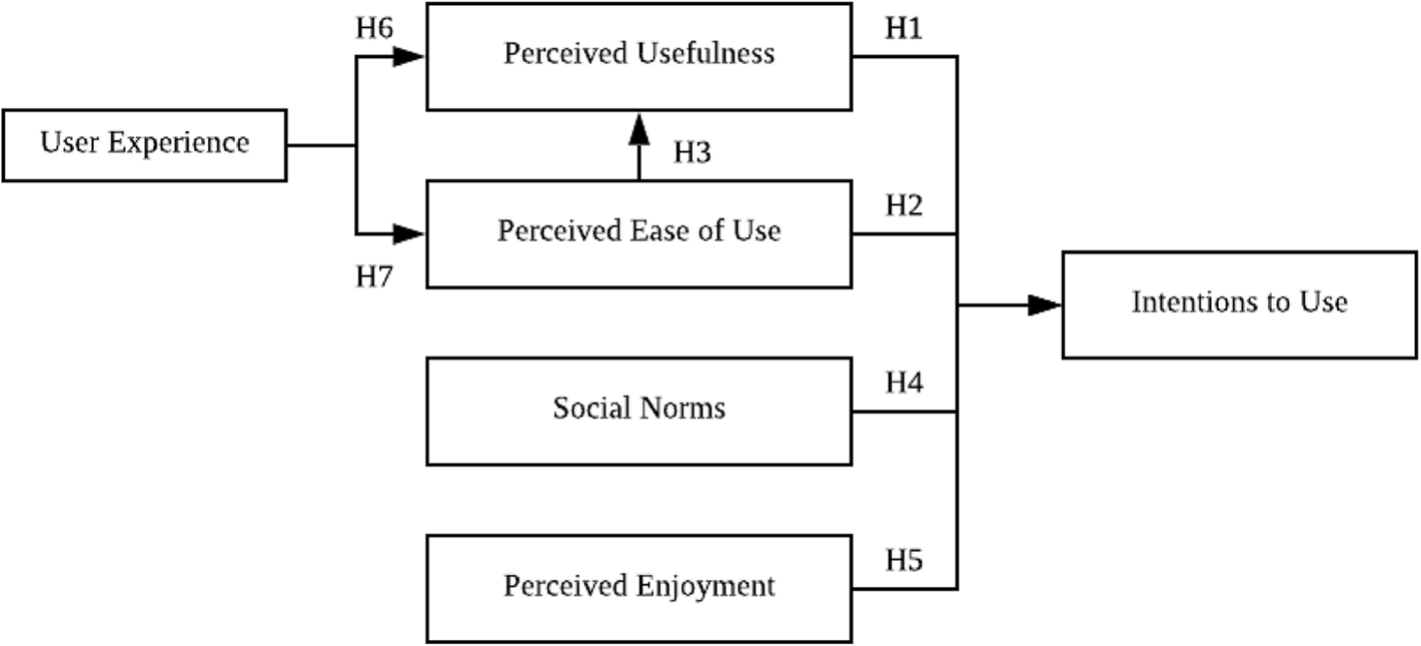
Research model for the study. This figure describes the research model developed for the study based on TAM and reviewed literature. H1-H7 are hypotheses 1~7, which describe the influence of one variable on the other

We composed our research model based on the TAM and a literature review as depicted in Fig. 1. This study attempted to explore the acceptance of the VR among an older population while mitigating problems of aging.

## Methodology

### Study setting and participants

The study was conducted among 30 participants of an older age group (six males and 24 females).

Participants were included under the following criteria: 1) aged 60 years and above, 2) visited the Taipei Medical University (TMU) aging center, and 3) were willing to participate in the study components.

### Data collection

A signed consent form was received from each participant after explaining to them the objective of the study, which was carried out from March to May 2018. Mandarin Chinese versions of the questionnaire and consent form were filled out by participants. The questionnaire is given in Additional file 1 (based on the TAM and SNs, UE, and PE from Venkatesh). The questionnaire was used to collect participants’ perceptions about their acceptance of VR. Six sets of questions were clustered under the variables of PU, PEOU, PE, SN, UE, and IU based on a literature review.

We used the Vive htc VR system for the study. These devices contained nine different applications (Appendix) that encouraged physical activity, motivated, and provided entertainment to older people. We ensured that participants accessed the different VR applications at least once during the study period. Participants were asked to use VR for 15 min twice a week for 6 weeks and respond to the questionnaire in Additional file 1 at the end of the study. A 5-point Likert scale was used to score the questions, in which participants were asked to agree or disagree based on a ranking from 5 to 1, where 5 indicated strong agreement, 3 indicated a neutral opinion, and 1 indicated strong disagreement. Information obtained from respondents was centered on their experience and perceptions while using VR. The study was approved by the ethics committee of the Taipei Medical University-Joint Institutional Review Board.

### Data analysis

Researchers analyzed the data using SPSS vers. 20 (SPSS, Chicago, IL, USA). Pearson’s product moment correlation was conducted to examine the criterion validity of the questionnaire. Cronbach’s alpha reliability analysis was used to test the internal consistency of the questionnaire items. A linear regression analysis was utilized to identify relationships among the variables: PU, PEOU, UE, SNs, PE, and IU. A mediation analysis was conducted to determine the mediating effect of different variables in the questionnaire.

## Results

### Characteristics of participants

Table 1 shows characteristics of the 30 older participants. Of these, six (20%) were males, and 24 (80%) were females, with the highest percentage of participants aged 70~75 years.

**Table 1:** Characteristics of participants

Age (years)	Male 6 (20%)	Female 24 (80%)	Total (%) *n* = 30
60~65	1	7	8 (26.7%)
65~70	3	4	7 (23.3%)
70~75	1	7	8 (26.7%)
75~80	1	3	4 (13.3%)
80~85	0	2	2 (6.7%)
85~90	0	0	0
> 90	0	1	1 (3.3%)

**Fig. 2:**
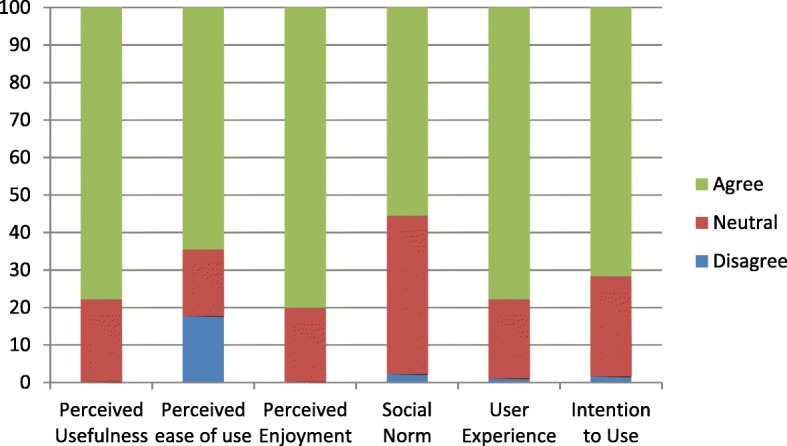
Summary of responses of participants. This figure explains the graph of the responses of the participants in terms of percentage

Figure 2 shows a summary of the responses of the 30 older participants. They were asked about the PU of VR, and 77.8% agreed that VR helped raise their mood, and entertain and keep them motivated to do their daily activities. Participants who believed that VR was easy to use accounted for 64.4, and 80% of participants found it enjoyable. For subjective norms with regards to the support of the use of VR by family, friends, and caretakers, 55.6% agreed, and 77.8% of users expressed a positive experience. Around 71.7% of participants intended to use VR in the future.

### Summary statistics of variables

Summary statistics and frequency distributions of all the variables are shown in Table 2, with a mean range of 3.27~4.07, and standard deviation of ≤0.661.

**Table 2:** Summary statistics and frequency distributions of variables in the questionnaire

Variable description	Mean	Std. deviation (SD)
Perceived usefulness (PU)		
PU1	3.87	0.571
PU2	3.80	0.610
PU3	4.07	0.583
Perceived ease of use (PEOU)		
PEOU1	3.87	0.571
PEOU2	3.27	0.556
PEOU3	3.67	0.583
Social norms (SNs)		
SN1	3.43	0.626
SN2	3.77	0.626
SN3	3.67	0.661
User experience (UE)		
UE1	4.07	0.583
UE2	3.77	0.626
UE3	3.83	0.592
Intention to use (IU)		
IU1	3.63	0.615
IU2	3.90	0.607

### Validity testing of the questionnaire

The validity coefficient and *p* values of items in the questionnaire are shown in Table 3.

**Table 3:** Criterion validity test of the questionnaire with Pearson’s product-moment correlations

Item in the questionnaire	rxy	*p* value
VR is useful to me for entertainment. (PU1)	0.727	0.000
VR improves engagement and motivates daily activities. (PU2)	0.706	0.000
VR is an efficient tool to raise my mood. (PU3)	0.590	0.001
It is easy for me to become skillful at using VR. (PEOU1)	0.752	0.000
Learning to operate VR was easy for me. (PEOU2)	0.678	0.000
Overall I find it easy to use VR. (PEOU3)	0.797	0.000
I find VR very attractive to use. (PE1)	0.842	0.000
I enjoy using VR. (PE2)	0.791	0.000
I have fun when I use VR. (PE3)	0.813	0.000
My family members think I should use VR. (SN1)	0.787	0.000
People who are friends and acquaintances have influence on my intention to use VR. (SN2)	0.842	0.000
People who take care of me encourage me to use VR. (SN3)	0.629	0.000
VR will give me new experiences. (UE1)	0.526	0.003
VR was comfortable to use. (UE2)	0.829	0.000
Overall, I had a positive experience when using VR. (UE3)	0.829	0.000
In the future, I intend to use the device for mental relaxation. (IU1)	0.813	0.000
In the future, VR will help keep my mind sharp and alert. (IU2)	0.838	0.000

VR, virtual reality

The critical value of Pearson’s product-moment was 0.361, according to the table of critical values [48], for 30 respondents. As shown in Table 3, for all our variables, the r table product moment (rxy) was > 0.361 for *p* values of < 0.05.

### Cronbach’s α reliability analysis

Cronbach’s α reliability analysis was conducted to assess the internal consistency and reliability of the variables in the study (Table 4).

**Table 4:** Cronbach’s α values

Item	Cronbach’s α
Perceived usefulness	0.922
Perceived ease of use	0.925
Perceived enjoyment	0.903
Social norms	0.910
User experience	0.913
Intention to use	0.899

### Hypothesis testing using a linear regression analysis

Seven hypotheses were formulated based on the initial TAM model and a literature review. Each hypothesis was tested for significance based on the regression statistics. Table 5 presents the summary of the regression data for each hypothesis. Larger beta values were observed for larger *t* values and smaller *p* values across all hypotheses.

**Table 5:** Regression statistics for the formulated hypotheses

Hypothesis	Independent variable	Dependent variable	Un-standardized coefficients		*F*	*t*	*p*	*R* ^2^	Hypothesis supported?
			β	Standard Error					
H1	PU	IU	0.625	0.125	25.205	5.020	0.000	0.474	Yes
H2	PEOU	IU	3.113	0.527	34.920	5.909	0.000	0.555	Yes
H3	PEOU	PU	2.523	0.727	12.058	3.472	0.002	0.301	Yes
H4	SNs	IU	0.717	0.131	29.845	5.463	0.000	0.516	Yes
H5	PE	IU	0.784	0.095	67.870	8.238	0.000	0.708	Yes
H6	UE	PU	2.065	0.439	22.137	4.705	0.000	0.442	Yes
H7	UE	PEOU	0.401	0.103	15.170	3.895	0.001	0.351	Yes

PU, perceived usefulness; IU, intention to use; PEOU, perceived ease of use; SNs, social norms; PE, perceived enjoyment; UE, user experience

All the hypotheses are detailed as below:

- H1: For H1, the regression analysis gave a *p* value of 0.000, which indicated that PU had a significant influence on IU. *R*^2^ for the regression equation was 0.474, indicating that the predictor factor PU explained 47.4% of IU. The un-standardized β coefficient indicated that for every unit of increase in PU, a 0.625 increase in the units of IU was predicted.
- H2: For H2, the regression analysis gave a *p* value of 0.000, which indicated that PEOU had a significant influence on IU. *R*^2^ for the regression equation was 0.555, indicating that the predictor factor PEOU explained 55.5% of IU. The un-standardized β coefficient indicated that for every unit of increase in PU, a 3.113 increase in the units of IU was predicted.
- H3: For H3, the regression analysis gave a *p* value of 0.002, which indicated that PEOU had a significant influence on PU. *R*^2^ for the regression equation was 0.301, indicating that the predictor factor PEOU explained 30.1% of PU. The un-standardized β coefficient indicated that for every unit of increase in PEOU, a 2.523 increase in the units of PU was predicted.
- H4: For H4, the regression analysis gave a *p* value of 0.000, which indicated that SNs had a significant influence on IU. *R*^2^ for the regression equation was 0.516, indicating that the predictor factor PEOU explained 51.61% of IU. The un-standardized β coefficient indicated that for every unit of increase in PEOU, a 0.717 increase in the units of IU was predicted.
- H5: For H5, the regression analysis gave a *p* value of 0.000, which indicated that PE had a significant influence on IU. *R*^2^ for the regression equation was 0.708, indicating that the predictor factor PE explained 70.8% of IU. The un-standardized β coefficient indicated that for every unit of increase in PE, a 0.784 increase in the units of IU was predicted.
- H6: For H5, the regression analysis gave a *p* value of 0.000, which indicated that UE had a significant influence on PU. *R*^2^ for the regression equation was 0.442, indicating that the predictor factor UE explained 44.2% of IU. The un-standardized β coefficient indicated that for every unit of increase in UE, a 2.065 increase in the units of PU was predicted.
- H7: For H7, the regression analysis gave a *p* value of 0.001, which indicated that UE had a significant influence on PEOU. *R*^2^ for the regression equation was 0.351, indicating that the predictor factor UE explained 35.1% of PEOU. The un-standardized β coefficient indicated that for every unit of increase in UE, a 0.401increase in the unit of PEOU was predicted.

### Mediation analysis

Table 6 describes mediating factors of possible pathways, using a bootstrap analysis method.

**Table 6:** Bootstrap analysis of the mediation effect

No	Path A	Path B	Path C′	Mediating factor	Mediation effect (full/partial)	Indirect effect (value)	*p* value	95% CI lower	95% CI upper
1	UE ➔ PEOU	PEOU ➔ PU	UE ➔ PU	PEOU	Partial	0.1305	0.2054	- 0.0066	0.3072
2	PEOU ➔ PU	PU ➔ SNs	PEOU ➔ SNs	PU	Partial	0.1051	0.1161	- 0.0442	0.3480
3	PU ➔ SNs	SNs ➔ IU	PU ➔ IU	SN	Full	0.3124	0.0142	0.0713	0.8124
4	PEOU ➔ SNs	SNs ➔ IU	PEOU ➔ IU	SN	Partial	0.1962	0.0534	- 0.0373	0.4613
5	PU ➔ PE	PE ➔ IU	PU ➔ IU	PE	Full	0.5064	0.0005	0.2799	0.8206
6	PEOU ➔ PE	PE ➔ IU	PEOU ➔ IU	PE	Partial	0.6427	0.0005	0.3372	1.3620
7	PEOU ➔ PU	PU ➔ IU	PEOU ➔ IU	PU	Partial	0.1575	0.0263	0.0558	0.3539

CI, confidence interval; UE, user experience; PEOU, perceived ease of use; PU, perceived usefulness; SNs, social norms; IU, intention to use

**Fig. 3:**
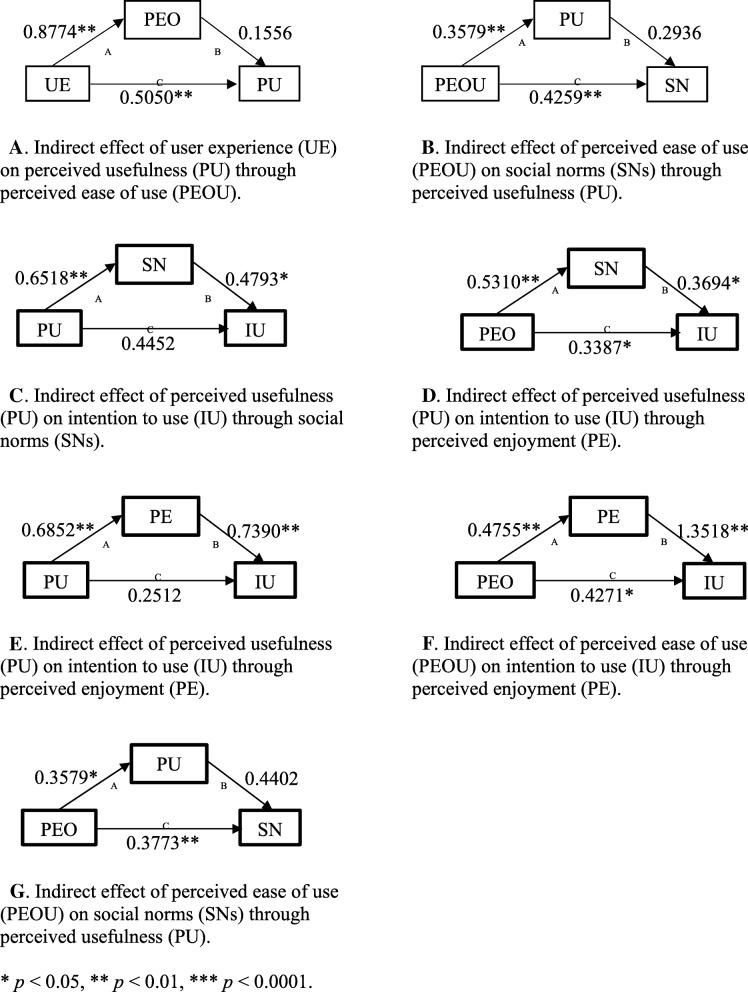
Models of mediating effects for different variables through pathways **a**, **b**, and **c**′. This figure describes the different models for mediating effects among different variables

Figure 3 describes models of the mediation effect of different variables through pathways A, B, and C′. In models 3C and 3E, paths A and B were significant, and C was not significant. Table 5 indicates that H1 was significant, which was not supported when mediation was used. Thus, SNs provided a strong mediating effect for determining the influence of PU on IU, and PE provided a strong mediating effect for determining the influence of PU on IU. Models 3F and 3G indicated that paths A, B, and C were significant; however, the mediators did not give significant effects for the models.

## Discussion

In this study, based on the TAM and a literature review, a research model was developed to determine factors influencing the intention to use VR among an older population.

Validity and reliability tests were conducted to ensure that the results were reliable and consistent. A criterion validity test was conducted with Pearson’s product-moment correlation. The r table product moment was < 0.361 for all of our variables; this value is from a table of critical values of the Pearson product-moment [48]. Furthermore, *p* values were all < 0.05, which implies that all of the variables were valid. According to Tavakol and Dennik [49], a Cronbach’s alpha value of > 0.7 is considered an acceptable value in terms of the reliability level. Cronbach’s α values for all variables in our study were > 0.70, which confirmed the internal reliability (Table 2). Linear regression statistics suggested that all of the formulated hypotheses were supported. SNs and perceived experience showed significant mediating effects in determining the influence of PU on the IU VR.

We conducted the study on the usefulness and acceptance of VR among an aged population. Participants agreed to a large extent to the PU, PE, and their experience of using VR. The PU and PEOU were seen to predict the IU VR. This suggests that older people consider using technology which is easy to use and also based on its usefulness. Our results are consistent with Davis’ postulation that users tend to adopt a technology based on the tasks it can perform for them and also on the level of difficulty associated with its usage [36, 38]. Similarly, SNs are seen to have a direct effect on the IU VR, which was also seen in the case of other technologies previously tested [41, 42, 50]. PE is seen as a more-significant predictor of IU compared to PU in our study, suggesting that older people regard the level of enjoyment as an important factor [51]. This was also determined by Van der Heiden [43]. User experience was seen to have a significant effect on the PU and PEOU. This implies that elderly users regard the experience as an important determinant of the usefulness of and ease of using VR.

PU, PEOU, and PE are among important factors to be considered when developing VR applications for use among elderly populations. Older people seemed to enjoy VR; however, poor health may prevent such new experiences, which necessitates one consider their preferences and remove barriers that can limit the use and enjoyment of VR [52].

VR technology can be introduced as one of the devices that can motivate older people to be more physically active [19, 20]. Older people found it useful in motivating them in their daily activities. In addition to contemporary comfort and ease of using VR, older people also intended to use it for mental relaxation in the future. VR was comfortable and provided a new and positive experience for them, which was also consistent with a previous study [53].

VR equipment is expensive if the device includes more features like haptic feedback, thus limiting its adoption [54]. One more limitation of VR equipment and programs is that if used for an extended period, it may cause a dizzy feeling in older adults and in turn decrease personal interactions and conversation among older people and their families [53]. Bearing in mind these factors, we would like to encourage older adults to willingly participate in using VR applications by understanding its uses as well as considering its limitations [55]. Our study provides an understanding of perceptions and preferences of the elderly towards the use of VR. In addition, it also indicates positive perceptions of the elderly towards the usefulness of VR to support aging.

Despite providing meaningful insights into the adoption and acceptance of VR among older people, our study also had some limitations. First, the small number of participants in the study limited the broader perspectives that could be accessed from larger numbers of respondents. In the future, adding more participants to the study and conducting the study for a longer duration would help ensure feedback from a larger population. Also, we would also select a broader range of VR programs including a variety of topics in order to respond to older adults’ interests and preferences. Second, our study included VR apps which encouraged individual participation. Future consideration of VR apps that could allow participation in groups and competition within specific programs could help promote social interactions in future versions of the study.

## Conclusions

Our study demonstrates the use and acceptance towards the adoption of VR among older population. Perceptions that VR was useful, was easy to use, and provided an enjoyable experience showed the positive attitudes of older adults toward adopting this new technology. Future developments could be made considering these perceptions among elderly populations.

### Additional file

Additional file 1: Acceptance of the Virtual Reality (VR) Experience among the Elderly: Questionnaire. (DOCX 16 kb)

## Data Availability

The data analyzed during the current study are available from the corresponding author on reasonable request.

## Appendix

Virtual Reality applications used during the study

1. The Lab
2. Everest VR
3. The Body VR: Journey Inside a Cell
4. To The Top
5. Waltz of the Wizard
6. Google Earth VR
7. Found
8. Sparc
9. Final Soccer VR

## Abbreviations

H1~7: Hypotheses 1~7
IU: Intention to use
PE: Perceived enjoyment
PEOU: Perceived ease of use
PI: Physical inactivity
PU: Perceived usefulness
SNs: Social norms
TAM: Technology Acceptance Model
TMU: Taipei Medical University
TRA: Theory of Reasoned Action
VR: Virtual reality
WHO: World Health Organization
