# Supplementary material for: Virtual reality among the elderly: a usefulness and acceptance study from Taiwan
Source: BMC Geriatr. 2019 Aug 19;19:223. doi: 10.1186/s12877-019-1218-8 (PMC6699111; doi:10.1186/s12877-019-1218-8)
Supplement: Supplementary file 1 — Acceptance of the Virtual Reality (VR) Experience among the Elderly: Questionnaire. (DOCX 16 kb) [file 12877_2019_1218_MOESM1_ESM.docx]

**Appendix 1**

**Acceptance of the Virtual Reality (VR) Experience among the Elderly: Questionnaire**

- **Perceived usefulness:**
  1. VR is useful to me for entertainment.
     - Strongly disagree
     - Disagree
     - Neutral
     - Agree
     - Strongly agree
  2. VR improves engagement and motivates my daily activities.
     - Strongly disagree
     - Disagree
     - Neutral
     - Agree
     - Strongly agree
  3. VR is an efficient tool to raise my mood.
     - Strongly disagree
     - Disagree
     - Neutral
     - Agree
     - Strongly agree
- **Perceived ease of use**
  1. It is easy for me to become skillful at using VR.
- Strongly disagree
- Disagree
- Neutral
- Agree
- Strongly agree
  1. Learning to operate VR was easy for me.
     - Strongly disagree
     - Disagree
     - Neutral
     - Agree
     - Strongly agree
  2. Overall I find it easy to use VR.
     - Strongly disagree
     - Disagree
     - Neutral
     - Agree
     - Strongly agree
- **Perceived enjoyment**
  1. I find VR very attractive to use.
     - Strongly disagree
     - Disagree
     - Neutral
     - Agree
     - Strongly agree
  2. I enjoy using VR.
     - Strongly disagree
     - Disagree
     - Neutral
     - Agree
     - Strongly agree
  3. I have fun when I use VR.
     - Strongly disagree
     - Disagree
     - Neutral
     - Agree
     - Strongly agree
- **Subjective norms**
  1. My family members think I should use VR.
     - Strongly disagree
     - Disagree
     - Neutral
     - Agree
     - Strongly Agree
  2. People who are friends and acquaintances have influence on my intention to use VR.
     - Strongly disagree
     - Disagree
     - Neutral
     - Agree
     - Strongly agree
  3. People who take care of me encourage me to use VR.
     - Strongly disagree
     - Disagree
     - Neutral
     - Agree
     - Strongly agree
- **User experience**
  1. VR will give me new experiences.
     - Strongly disagree
     - Disagree
     - Neutral
     - Agree
     - Strongly agree
  2. VR was comfortable to use.
     - Strongly disagree
     - Disagree
     - Neutral
     - Agree
     - Strongly agree
  3. Overall, I had a positive experience when using VR.
     - Strongly disagree
     - Disagree
     - Neutral
     - Agree
     - Strongly agree
- **Intentions to use**
  1. In the future, I intend to use the device for mental relaxation.
     - Strongly disagree
     - Disagree
     - Neutral
     - Agree
     - Strongly agree
  2. In the future, VR will help keep my mind sharp and alert.
     - Strongly disagree
     - Disagree
     - Neutral
     - Agree
     - Strongly agree
